# Unbiased Phenotype-Based Screen Identifies Therapeutic Agents Selective for Metastatic Prostate Cancer

**DOI:** 10.3389/fonc.2020.594141

**Published:** 2021-03-02

**Authors:** Ivy Chung, Kun Zhou, Courtney Barrows, Jacqueline Banyard, Arianne Wilson, Nathan Rummel, Atsushi Mizokami, Sudipta Basu, Poulomi Sengupta, Badaruddin Shaikh, Shiladitya Sengupta, Diane R. Bielenberg, Bruce R. Zetter

**Affiliations:** ^1^Vascular Biology Program, Boston Children’s Hospital, Boston, MA, United States; ^2^Department of Surgery, Harvard Medical School, Boston, MA, United States; ^3^Center for Veterinary Medicine, U.S. Food and Drug Administration, Washington, DC, United States; ^4^Department of Integrative Cancer Therapy and Urology, Kanazawa University Graduate School of Medical Sciences, Kanazawa, Japan; ^5^Laboratory for Nanomedicine, Department of Medicine, Brigham and Women’s Hospital, Boston, MA, United States

**Keywords:** metastasis, prostate, carcinoma, bone, drug-screen

## Abstract

In American men, prostate cancer is the second leading cause of cancer-related death. Dissemination of prostate cancer cells to distant organs significantly worsens patients’ prognosis, and currently there are no effective treatment options that can cure advanced-stage prostate cancer. In an effort to identify compounds selective for metastatic prostate cancer cells over benign prostate cancer cells or normal prostate epithelial cells, we applied a phenotype-based *in vitro* drug screening method utilizing multiple prostate cancer cell lines to test 1,120 different compounds from a commercial drug library. Top drug candidates were then examined in multiple mouse xenograft models including subcutaneous tumor growth, experimental lung metastasis, and experimental bone metastasis assays. A subset of compounds including fenbendazole, fluspirilene, clofazimine, niclosamide, and suloctidil showed preferential cytotoxicity and apoptosis towards metastatic prostate cancer cells *in vitro* and *in vivo*. The bioavailability of the most discerning agents, especially fenbendazole and albendazole, was improved by formulating as micelles or nanoparticles. The enhanced forms of fenbendazole and albendazole significantly prolonged survival in mice bearing metastases, and albendazole-treated mice displayed significantly longer median survival times than paclitaxel-treated mice. Importantly, these drugs effectively targeted taxane-resistant tumors and bone metastases – two common clinical conditions in patients with aggressive prostate cancer. In summary, we find that metastatic prostate tumor cells differ from benign prostate tumor cells in their sensitivity to certain drug classes. Taken together, our results strongly suggest that albendazole, an anthelmintic medication, may represent a potential adjuvant or neoadjuvant to standard therapy in the treatment of disseminated prostate cancer.

## Introduction

In the United States, prostate cancer is one of the most prevalent cancers and the second leading cause of cancer death in males (1). Mortality from prostate cancer is largely restricted to patients with disseminated metastases in bone or in other distal sites. Despite a high five-year relative survival rate approaching 100% for men with localized disease, the rate in patients with disseminated cancer is only 31% (2). Nearly one quarter of all prostate cancer patients will develop metastatic disease following surgery or radiation therapy (3–5). Although androgen ablation is often effective as an initial treatment for recurrent prostate cancer, the disease almost inevitably progresses to a hormone-refractory state. Men with hormone-refractory disease have a poor prognosis, with median survival times ranging from 9 to 30 months (6, 7). In addition, substantial morbidity is associated with this disease and can include pain, fractures, spinal cord compression, and ultimately, death. The management of hormone refractory metastatic prostate cancer has been limited to palliation of symptoms due to a lack of effective treatments. Taxane-based chemotherapy has improved survival in some men with metastatic disease (8, 9). Although most patients initially respond to docetaxel, the majority of these cancers eventually develop insensitivity, resulting in a more aggressive, chemotherapy-resistant disease (10). There is an urgent need to find alternative treatments designed to prolong survival of patients with metastatic prostate cancer.

It is widely observed that metastatic lesions in many cancers respond poorly to existing therapies, including chemo- and radiation therapy. The reasons for this are unclear but may be due to the extent of the metastatic burden or due to intrinsic properties of metastatic cells that render them refractory to treatments that are effective against less aggressive tumors. Studies have demonstrated that these metastatic tumor cells may acquire stem cell-like properties including expression of certain cell-surface markers (11, 12), and therefore exhibit increased resistance to chemotherapeutic agents and ionizing radiation (13). Likewise, during the metastatic cascade, these cells may also acquire susceptibilities to certain agents. We hypothesized that such vulnerabilities could be targeted in developing effective agents for treatment of metastatic prostate cancer.

To test this hypothesis, the relative differential cytotoxicity of multiple panels of highly metastatic and poorly metastatic prostate cancer cells were tested using a library of 1120 drugs, most of which are Food and Drug Administration (FDA)-approved. Intriguingly, several drugs demonstrated increased cytotoxicity against tumor cells with high metastatic potential in *both in vitro* and *in vivo* assays. Because these drugs are already used in humans for different purposes, such newly discovered agents could be quickly entered into human clinical efficacy studies using the existing drug administration regimen or be improved based on the known pharmacokinetic and pharmacodynamic profiles. Our work demonstrates that it is possible to conduct a screen for drugs that can effectively treat metastatic tumors and further suggests that these newly identified agents may be examples of a “repurposed drug” approach in drug development.

## Materials and Methods

### Chemicals and Reagents

**Figure 1:**
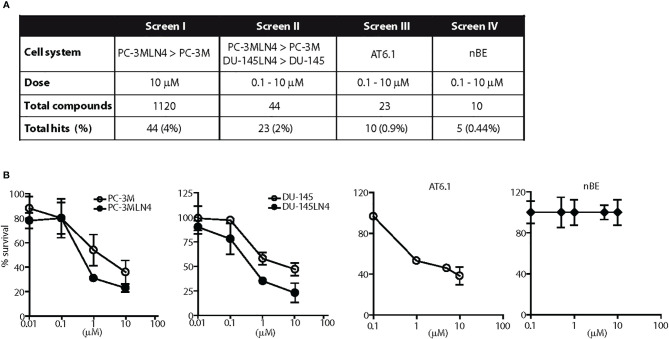
Screen for agents with a selective cytotoxicity for highly metastatic prostate cancer cells. **(A)** Diagram illustrates the results of four independent and sequential screens for cytotoxic agents against metastatic prostate cancer cells. One thousand one hundred-twenty drugs were tested in at least two concentrations. **(B)** Representative dose response testing fenbendazole in prostate cancer and normal prostate cells for 48 h. Fenbendazole exhibited: 1) greater cell killing in highly metastatic PC-3MLN4 compared to less metastatic PC-3M cells at 10 μM, p < 0.01 (Screen I); 2) greater cell killing in both highly metastatic PC-3MLN4 and DU-145LN4 cells compared to the less metastatic counterparts, p < 0.01 for cell killing at 1 and 10 µM (Screen II); 3) a greater than 40% cell killing in AT6.1 cells, p < 0.001 for dose above 0.1 µM (Screen III); and 4) minimal cytotoxicity in normal prostate epithelial nBE cells, p > 0.05 (Screen IV). Data shown are mean ± 95% confidence interval, N=4 per group.

**Figure 2:**
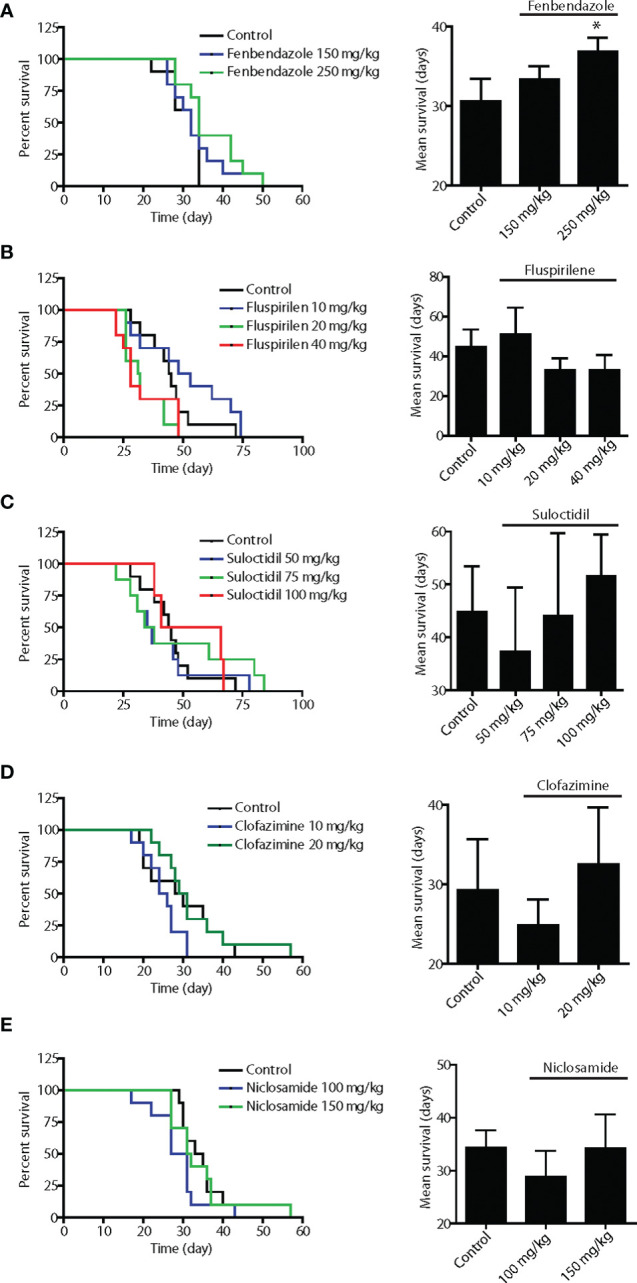
*In vivo* screening of the five lead hits in survival assays using disseminated Dunning rat AT6.1 prostate carcinoma cells. To generate pre-existing micrometastatic lesions, AT6.1 Dunning rat prostate carcinoma cells were injected intravenously into nude mice five days prior to treatment. Fenbendazole **(A)**, fluspirilene **(B)**, suloctidil **(C)**, clofazimine **(D)** and niclosamide **(E)** were given three times a week *via* intraperitoneal injection until signs of morbidity were observed. The drugs were solubilized in DMSO and diluted with saline prior to injection. Shown is Kaplan-Meier survivals curves (left) and mean survival (right) of mice treated with either vehicle (control) or agents. Data shown are mean ± 95% confidence interval, N=10 per group, *p < 0.05.

**Figure 3:**
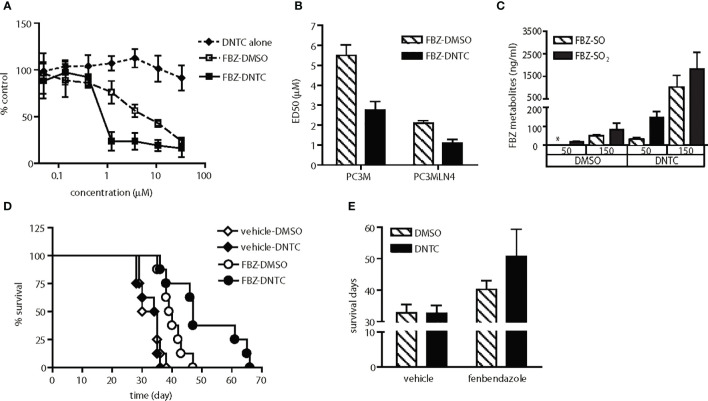
Enhanced anti-tumor activity of fenbendazole was observed upon improvement in solubility and bioavailability. **(A)** Fenbendazole (FBZ) cytotoxicity in PC-3MLN4 cells is shown for drugs solubilized in either DNTC or DMSO. DNTC itself exhibits minimal cytotoxicity. FBZ-DNTC and FBZ-DMSO were significantly more cytotoxic than DNTC alone, p<0.001 for doses ≥1 µM. **(B)** Comparison of ED50 of FBZ when formulated in the two different vehicles, DMSO and DNTC; p<0.001. **(C)** Measurement of FBZ and its active metabolites (FBZ-SO and FBZ-SO_2_) in the mouse plasma collected after one injection. FBZ was not detected in all samples. *, under limit of detection. **(D)** AT6.1 cells were inoculated intravenously 5 days prior to treatment of 100 mg/kg fenbendazole either in DMSO or DNTC, given 3 times a week until signs of morbidity were observed. Shown are Kaplan Meier survival curves of mice in each group. N=8 per group, p<0.001 in FBZ-DMSO vs. FBZ-DNTC. **(E)** Average of survival days for each treatment group from panel **(D)** Data shown are mean ± 95% confidence interval, N=8 per group, p<0.001.

**Figure 4:**
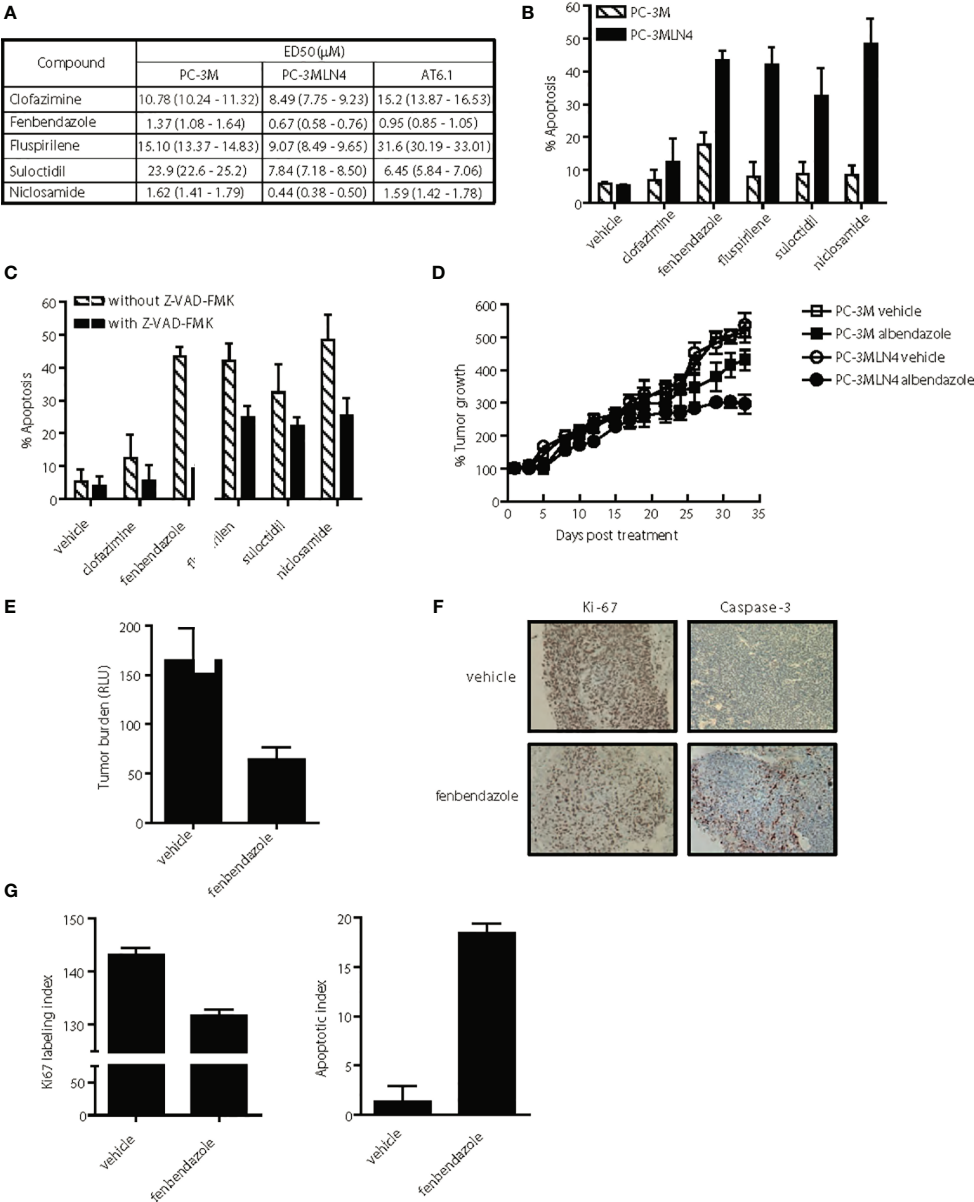
Selective anti-tumor activity of lead hits on highly aggressive, metastatic prostate cancer cells. **(A)** ED50 of agents for highly metastatic (PC-3MLN4 and AT6.1) and less metastatic (PC-3M) prostate cancer cells after 72 h treatment, as determined using Cyquant proliferation assay. Data shown are mean ± 95% confidence interval, N=3 per group. **(B)** Percent apoptosis was determined by annexin V-stained cells after 72 hr treatment with 1 μM drug. Data shown are mean ± 95% confidence interval, N=4 per group. PC-3MLN4 were significantly (p < 0.01) more apoptotic than PC-3M when treated with fenbendazole, fluspirilene, suloctidil, and niclosamide. **(C)** Drug-induced apoptosis in PC-3MLN4 was significantly (p < 0.05) reduced by treatment with 20 µM caspase-3 inhibitor (Z-VAD-FMK) for fenbendazole, fluspirilene, and niclosamide. **(D)** PC-3M and PC-3MLN4 subcutaneous tumors were treated with 100 mg/kg fenbendazole, three times a week for five weeks. Percent tumor growth was calculated from the tumor volume measured on first day of treatment. Data shown are mean ± 95% confidence interval, N=10 per group. Tumor growth was significantly (p < 0.01) more reduced in PC-3MLN4 treated with FBZ (44.9%) than in PC-3M treated with FBZ. **(E–G)** AT6.1 cells were inoculated *via* tail vein 5 days prior to treatment with 100 mg/kg fenbendazole, given 3 times a week until day 22 when signs of morbidity were observed in the control group. **(E)** Secreted Gaussia luciferase activity in the peripheral blood, indicative of tumor burden in the mice was measured on the day of harvest, p < 0.001. **(F)** Representative staining of Ki-67 and activated caspase-3 on tumors in the lungs. **(G)** Ki-67 labeling and apoptotic index was determined using Imagescope software (Aperio). N=10 mice/group, p < 0.001.

**Figure 5:**
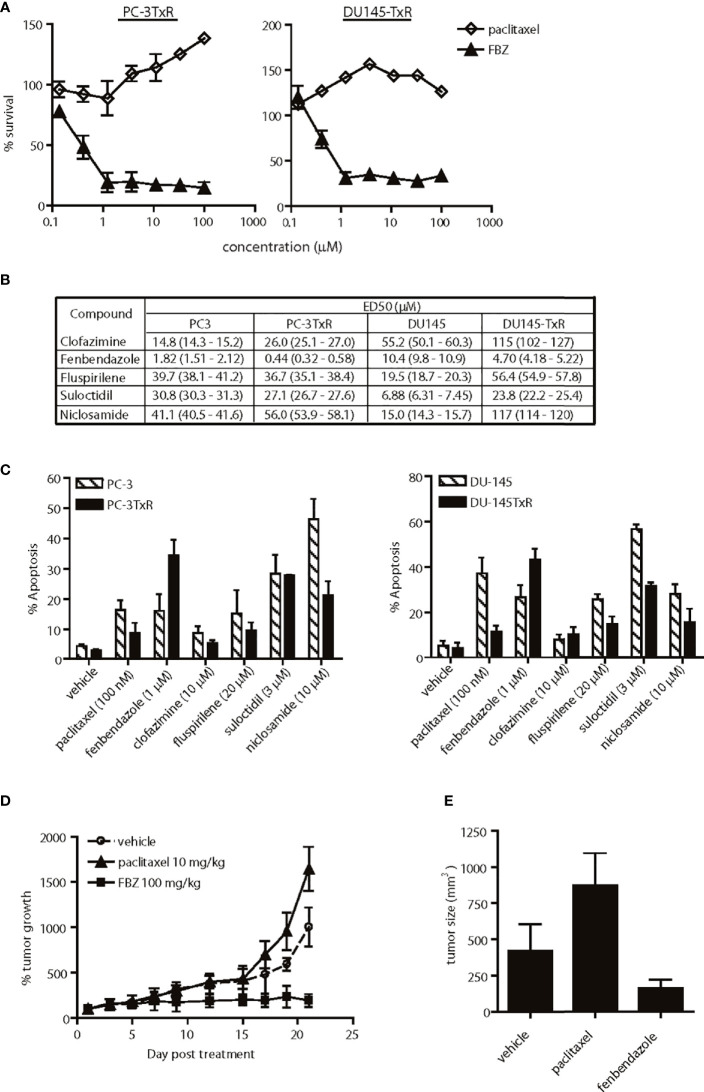
Effects of lead agents in paclitaxel-resistant prostate cancer cells. **(A)** Cytotoxic effects of paclitaxel and fenbendazole (FBZ) in paclitaxel-resistant prostate cancer cells. FBZ-DNTC was significantly (p < 0.001) more cytotoxic than paclitazel alone in both cell lines for doses ≥1 µM. **(B)** Comparison of ED50 of agents in paclitaxel-sensitive and -resistant cell lines. **(C)** Percent apoptosis induced by each agent in paclitaxel-sensitive and -resistant cell lines, as measured by annexin V staining. Comparing the data in **(B, C)**, FBZ was the only drug significantly (p<0.05) more cytotoxic/apoptotic in both paclitaxel-resistant prostate cancer cell lines. **(D)** Relative growth of subcutaneous PC-3TxR tumors after treatment with DNTC alone, paclitaxel or FBZ in DNTC. Treatment was given three times per week for three weeks. FBZ significantly inhibited tumor growth compared to paclitaxel or vehicle, N=10 per group, p < 0.01. **(E)** Average of tumor size from each treatment group at the end of study. Data shown are mean ± 95% confidence interval, N=10 per group, p < 0.01.

**Figure 6:**
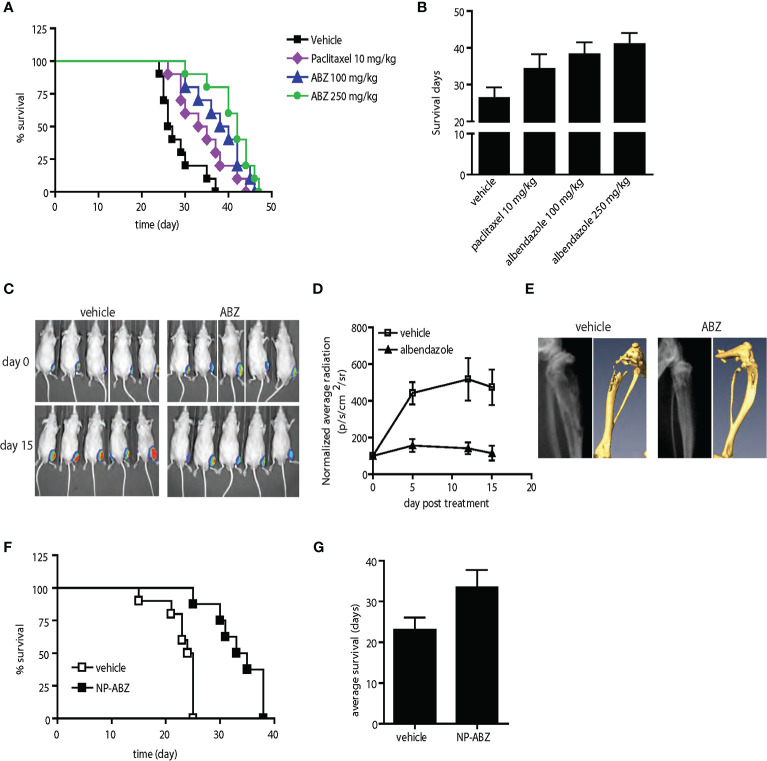
Anti-tumor activity of albendazole in comparison with paclitaxel and in experimental bone metastasis of prostate cancer *in vivo*. **(A, B)** AT6.1 cells were inoculated intravenously five days prior to treatment of either paclitaxel or albendazole (ABZ). Shown are Kaplan Meier survival curve **(A)** and the average survival days **(B)** for each treatment group; N=10 per group, p<0.001 in vehicle vs. paclitaxel, vehicle vs. ABZ 100mg/kg, vehicle vs. ABZ 250mg/kg and paclitaxel vs. ABZ 250mg/kg. **(C–E)** Bone lesions of PC-3MLN4 cells were generated by intraosseous injection into the tibia of mice, before treatment with vehicle or 100 mg/kg albendazole (ABZ), three times a week for two weeks. **(C)** Representative photos of luciferase signal detected in each treatment group at the beginning and end of study. **(D)** Mean of luciferase signals detected in mice during treatment, as a measurement of tumor burden. **(E)** Representative images from X-ray (left) and micro-CT (right) analysis showing differences in radiographical characteristics of the bone lesions. Data shown are mean ± 95% confidence interval, N=10 per group. **(F, G)** AT6.1 cells were inoculated intravenously five days prior to treatment of either vehicle or nanoparticle-albendazole (NP-ABZ). Shown are Kaplan Meier survival curve **(F)** and the average survival days **(G)** for each treatment group; N=10 per group, p < 0.001 in vehicle vs. NP-ABZ.

A customized drug library containing a total of 1,120 compounds was obtained from the Prestwick Chemical Library (Prestwick Chemical, Illkirch-Graffenstaden, France). All drugs in the drug library were solubilized in DMSO (**Figure 1**), subsequent experiments used other diluents (discussed below). The following chemicals were obtained from Sigma-Aldrich: fenbendazole (F5396), albendazole (A4673), fluspirilene (F100), clofazimine (C8895), niclosamide (N3510), suloctidil (S9384), tween-80 (P4780), N-methyl-2-pyrrolidone (NMP) (328634) and cremophor EL (Cr-EL) (C5135). Suloctidil and niclosamide were always prepared and used in DMSO (**Figures 2**, **4**, **5**). Fenbendazole, fluspirilene, and clofazimine were prepared in DMSO (**Figures 1**–**3**) or DNTC (**Figures 3**–**5**). Albendazole was prepared in DNTC or nanoparticles (PLGA) (**Figure 6**). Paclitaxel was obtained from Cytoskeleton (for *in vitro*) or Bristol-Myers Squibb (for *in vivo*). Gaussia luciferase system (LP-07) was purchased from Targeting Systems. Caspase inhibitor Z-VAD-FMK (03FK10901) and Matrigel (354323) were purchased from MP Biomedical and BD Biosciences, respectively.

### Cell Culture

PC3 and DU-145 were obtained from the American Type Culture Collection. PC-3M and PC-3MLN4 human prostate cancer cells were provided by I. Fidler, MD Anderson Cancer Center (14). AT6.1 rat prostate carcinoma cells were provided by J. Isaacs, Johns Hopkins University School of Medicine (15). NbE-1 rat prostate epithelial cells were provided by M. Freeman, Cedars-Sinai (16). PC-3TxR and DU-145TxR cells and the sensitive counterparts were provided by A. Mizokami (17). DU-145LN4 cells were created by serially cycling DU-145 cells or its derivatives from the prostate to draining lymph nodes in mice (18, 19). All PC-3 and DU-145 derivative cell lines were maintained in RPMI 1640 and DMEM, respectively, supplemented with 10% fetal bovine serum and 1% penicillin/streptomycin.

### Synthesis of Nanoparticles

Compounds were prepared either in 100% DMSO or in a mixture of DMSO, NMP, tween-80 and cremophor EL (DNTC) in 1:3:2:2 ratio. The resultant mixture was stable and was stored in room temperature. Nanoparticles were formulated using an emulsion-solvent evaporation technique, and PLGA-PEG conjugate was synthesized as described (20). PLGA (MW4.2 kD, 40 mg) and PLGA-PEG (10 mg) were dissolved in acetone and mixed with albendazole (5 mg). The solution was emulsified into a 2% aqueous solution of PVA (80% hydrolyzed, MW9-10kD) by slow injection with constant homogenization. NP size fraction was recovered by ultracentrifugation at 3,000 x g for 5 min and 90,000 x g for 2 h. The size distribution of nanoparticles was examined using a Malvern Zetasizer dynamic light scattering-system equipped with a He-Ne laser. The albendazole loading in the nanoparticles was determined by UV-VIS spectroscopy at 298 nm.

### Characterization of Nanoparticles

Albendazole NPs were suspended in 500 μL of AT6.1 cell lysate and sealed in a dialysis bag (MWCO ~ 1,000 Da). The dialysis bag was incubated in 1 mL of PBS buffer at room temperature with gentle shaking. Ten microliters of aliquot was extracted from the incubation medium at predetermined time intervals, dissolved in 90 μL DMF and released albendazole was quantified by UV-VIS spectroscopy at characteristic wavelength of albendazole, λ = 298 nm. After withdrawing each aliquot the incubation medium was replenished by 10 μL of fresh PBS.

### Survival Assay

Cell survival assay using Cyquant reagent (Invitrogen, C7026) was performed as described (19). Briefly, 1–4 x 10^5^ cells/mL were seeded in 96 well plates overnight, before treatment with either vehicle or drug for 48–72 h. Cells were harvested, washed and frozen at -80°C overnight. After thawing, Cyquant reagent was added and fluorescence intensity was measured in a spectrometer at 485 nm. Relative percent survival was calculated by dividing the reading from drug-treated cells by the reading from vehicle-treated cells. Median-dose effect analysis and ED50 calculation (50% reduction in cell survival) was performed using CompuSyn software (ComboSyn, Inc.), as described previously (21).

### Apoptosis Detection

Annexin V/7-amino-actinomycin D assay was performed following the manufacturer’s instructions (BD Pharmingen, 559763) and samples were analyzed by flow cytometry. Briefly, cells were treated for 72 h with either vehicle or compounds, with or without 20 µM z-VAD-FMK caspase inhibitors. Cells were trypsinized, washed and resuspended in assay binding buffer, labeled with annexin V and 7-amino-actinomycin before analysis by flow cytometry (BD FACScan). The apoptotic cell population was determined by the mean of positive staining of annexin V from three replicates. Data shown were representative of three independent experiments.

### Measurement of Benzimidazole Metabolites

Mice were given a bolus injection of 50 or 150 mg/kg drug prepared in DMSO or DNTC formulation before collection of plasma samples 8 h post injection. Benzimidazoles and their active metabolites (both sulfones and sulfoxides) in the plasma were extracted by liquid-liquid partition using potassium carbonate, DMSO, sodium metabisulfite and ethyl acetate, dried and analyzed by high performance liquid chromatography (HPLC) on a 150 x 4.6 mm Phenomenex Luna C18 5-µ column. Fenbendazole and its metabolites were detected using UV light (290 nm), while albendazole and its metabolites were detected using fluorescence (excitation at 290 nm; emission at 330 nm).

### *In vivo* Tumor Studies

#### Mice

All *in vivo* assays were performed in male Balb/c Nu/Nu mice (6–8 weeks old) purchased from Massachusetts General Hospital (Boston, MA) and housed in the Animal Resource at Children’s Hospital (ARCH) facility that is accredited by the American Association for Accreditation of Laboratory Animal Care (AAALAC). All experiments were conducted in accordance with the principles and procedures outlined in the NIH Guide for the Care and Use of Laboratory Animals and approved by an Institutional Animal Care and Use Committee (IACUC) at Boston Children’s Hospital.

#### Experimental Lung Metastasis

AT6.1 cells (10^4^) were injected *via* tail vein in HBSS. On day 5 post inoculation, treatment was begun and continued three times per week *via* intraperitoneal injection in 0.5 mL volume. Drugs were prepared in DMSO alone, DNTC vehicle or PLGA-PEG vehicle and diluted in normal saline prior to injection. Treatment was continued until the animals showed signs of morbidity defined by significant loss of body weight, difficulty in breathing and/or hunched posture.

#### Experimental Bone Metastasis

Luciferase-labeled PC-3MLN4 (2 x 10^6^) cells were injected directly into the tibia of mice as described (22). Bone lesions were monitored weekly by bioluminescent imaging (Xenogen IVIS™, Xenogen, CA). Two weeks post injection, mice with comparable tumor lesions were randomly divided into two groups for treatment of either vehicle or 100 mg/kg albendazole, given intraperitoneally three times per week for two weeks. At the end of the study, radiographical and anatomical changes of the bone lesions were obtained using X-ray (Faxitron X-Ray, IL) and micro-computed tomography (Siemens CAT II scanner, Siemens, NY).

#### Subcutaneous Tumors

Prostate tumor cells (2x10^6^) were mixed 1:1 with Matrigel and injected subcutaneously on the dorsal flank. When tumors reached approximately 100 mm^3^ in size, treatment began and continued three times a week as described above. Tumor volume was measured using a caliper and calculated with the formula: Volume = [Length x (width)^2^]/2 Relative tumor growth was determined by normalizing the tumor size at each time point with the initial size prior to the start of treatment.

### Blood Luciferase Assay

Blood Gaussia luciferase assay was performed as described (23).

### Immunohistochemistry

Tissues were collected and fixed in formalin before processing for paraffin embedding. Tissue sections were then stained for Ki-67 and caspase-3 as a service by the Dana Farber Cancer Institute/Harvard Cancer Center Research Pathology Core. The immunostainings were performed using Ki-67 antibody (DAKO, M7248) and caspase-3 antibody (Cell Signaling, 9664S), followed by detection methods using streptavidin HRP and rabbit ENVISION (DAKO), respectively. The immunostained slides were scanned using an Aperio CS Scanner (Vista, CA) at 20x magnification. Using Imagescope v10, at least 50 digital images of areas containing the tumor were randomly selected from 8 tissues to represent each treatment group. Ki-67 and caspase-3 staining intensities were scored using validated IHC nuclear image and color deconvoluted algorithms, respectively. Intensity was scored as 0 for absence of staining, 1+ for weak, 2+ for moderate, and 3+ for strong staining. Based on the percentage of these intensities, a score is determined with the formula: Score = (1.0 x %weak) + (2.0 x %moderate) + (3.0 x %strong). Both necrotic and peripheral tissue areas were excluded from the analysis.

### Statistical Analysis

All values are expressed as means ± 95% confidence interval. For comparisons between two groups, statistical significance was assessed with a two-tailed unpaired Student’s t test. The computations and graphs were performed and constructed with the GraphPad Prism 4.0 scientific graphing, curve fitting and statistics program (GraphPad Software, CA).

## Results

### Screening for Cytotoxic Agents Selective for Metastatic Prostate Cancer

In an unbiased fashion, the cytotoxic effects of 1,120 different compounds from a commercial chemical drug library were tested in prostate cancer cell lines in several phases of *in vitro* screening (**Figure 1A**). In the first phase (Screen I), drugs were selected based on preferential toxicity towards highly metastatic human prostate cancer cells PC-3MLN4 relative to the less aggressive counterpart, PC-3M (14). Cells were treated with a fixed dose of drug (10 µM) and analyzed for remaining viable cells after 48 h. A drug was considered a “hit” if it resulted in a) significantly greater growth inhibition in PC-3MLN4 than in PC-3M cells; or b) at least 80% growth inhibition in both cell lines. Only 4% of the drugs tested were identified as “hits” and were further taken into the second line of screening. In Screen II, drugs were tested over a range of concentrations for 48 h in two different human prostate cancer paired lines, PC-3M/PC-3MLN4 and DU-145/DU-145LN4 (18, 19). Positive “hits” from this screen exhibited more than 50% growth inhibition, preferential cytotoxicity to both metastatic cell lines, and a statistically significant difference (P<0.05) in sensitivity between the parental counterpart and metastatic variant for at least two of the concentrations tested. This screen resulted in 23 drug candidates (2% of the total library).

To further increase the stringency of our screening process, the positive candidates were next tested in a highly aggressive Dunning rat prostatic adenocarcinoma AT6.1 model (Screen III). AT6.1 cells develop lymph node and lung metastases when inoculated subcutaneously in rats or in immunocompromised mice (15, 24). In this screen, cells were treated with drugs over a range of concentrations for 48 h; drugs were chosen when they induced more than 40% growth inhibition in at least two concentrations tested.

To prepare for *in vivo* testing, the 10 drugs that passed through Screen III were assessed for minimal cytotoxic effects to normal prostate cells (>90% survival) using non-tumorigenic rat prostate epithelial NbE cells (Screen IV). From this screening sequence, we finally identified five candidate drugs: clofazimine, fenbendazole, fluspirilene, niclosamide and suloctidil that selectively exerted cytotoxic effects on all metastatic prostate cancer cell lines tested but not on normal rat epithelial cells. For example, treatment of 1 μM fenbendazole resulted in 69% (95% CI=67.04 to 70.96%) and 65% (95% CI=64.02 to 65.98%) growth inhibition in the highly metastatic PC-3MLN4 and DU-145LN4 cells, respectively, when compared to 46% (95% CI=42.08 to 49.92%) in poorly metastatic PC-3M cells and 49% (95% CI=47.04 to 50.96%) in DU-145 cells (**Figure 1B**). Fenbendazole at 1 μM concentration also exhibited cytotoxicity in the highly aggressive AT6.1 cells (53.2% growth inhibition, 95% CI=51.7 to 54.6%) but not in the normal prostate epithelial NbE cells (0% growth inhibition, 95% CI=−2 to 2%) (**Figure 1B**).

The anti-helminthic agents, fenbendazole and niclosamide, are widely used in treating tapeworm infections in veterinary medicine and in humans, respectively (25). Clofazimine is currently used for treatment of leprosy whereas flusprilene is used in the treatment of schizophrenia (26). Suloctidil, a vasodilator, was formerly used in the management of peripheral and cerebral vascular disorders (27). Identification of these agents in our multi-stage *in vitro* screen prompted us to investigate their potential anti-tumor activity *in vivo*.

### *In Vivo* Screening of Lead Drugs in an Experimental Lung Metastasis Survival Model

To effectively treat metastatic cancer, a drug must have the ability to slow the growth of pre-established metastatic colonies. Therefore, we employed an assay in which experimental metastases were established in the lungs of immunocompromised mice following tail-vein injection of Dunning rat AT6.1 prostate carcinoma. Drug treatment was started at a time point when colonies were observed in the lungs of test mice, generally at 5 days post-inoculation. Mice were given intraperitoneal injections of vehicle or drugs in several doses, three times per week until signs of morbidity were observed and euthanasia was necessary. The drugs were first solubilized in DMSO and diluted with saline prior to injection. The dose selection in rodent studies was based on previously published studies using these agents (27–31). The Kaplan-Meier survival curves of mice treated with fenbendazole, fluspirilene, suloctidil, clofazimine and niclosamide as well as the mean survival days of each group are shown in **Figure 2**. Except for fenbendazole at 250 mg/kg, all drugs showed little to moderate effect in improving the survival of mice with lung metastases. Compared to the vehicle-treated group (mean 30.6 days, 95% CI=29.1 to 33.1), mice receiving fenbendazole treatment showed a significant increase in survival times (mean 36.9 days, 95% CI=35.4 to 38.4; P=0.04) (**Figure 2A**). Fluspirilene at a lower dose (10 mg/kg) demonstrated a greater survival (mean 51.1 days, 95% CI=39.6 to 62.6 days vs. control mean 44.8 days, 95% CI=37.4 to 52.2 days) when compared to those with higher doses (20–40 mg/kg), suggesting that lower doses than 10 mg/kg may provide even greater survival benefit to these tumor-bearing mice (**Figure 2B**).

### Improved Drug Solubility for Delivering Anti-Tumor Agents *In Vivo*

We observed that these drugs (except for suloctidil) are highly hydrophobic and produce visible aggregates when diluted with saline. Because these aggregates can be observed in the peritoneum of treated mice during necropsy, we hypothesized that low drug delivery to metastatic lesions in the lung may explain the lack of anti-tumor activity observed in the animal studies. To improve systemic delivery of these agents, we determined that a combination of organic solvents including dimethyl sulfoxide (DMSO) and N-methyl-2-pyrrolidone (NMP) with surfactants Tween-80 and Cremophor EL (DNTC) in a 1:3:2:2 ratio provided improved solubility. This new DNTC vehicle did not cause significant toxicity *in vitro* in PC-3MLN4 cells (**Figure 3A**) or in PC3M cells (**Figure 4B**) or *in vivo* in PC-3M (**Figure 4D**) or AT6.1 (**Figures 4E–G**). Moreover, fenbendazole solubilized in DNTC showed increased cytotoxicity for PC-3M and PC-3MLN4 prostate cancer cells compared to those solubilized in DMSO (**Figures 3A, B**). There was ~2-fold reduction in ED50 (defined as the dose that kills 50% of the cell population) when tested in these cells; DMSO vs. DNTC, 5.5 μM (95% CI=5.3 to 5.7) vs. 2.76 μM (95% CI=2.6 to 2.9) in PC-3M and 2.1 μM (95% CI=2.05 to 2.15) vs. 1.1 μM (95% CI=1.03 to 1.17) in PC-3MLN4 cells, p<0.0001 (**Figure 3B**).

The bioavailability of the fenbendazole DNTC drug was analyzed *in vivo* by measuring the level of fenbendazole and its active metabolites, fenbendazole sulfone and sulfoxide, in plasma. Mice were injected intraperitoneally with a bolus dose of fenbendazole, formulated either in DNTC or DMSO, at 50 or 150 mg/kg (**Figure 3C**). At 8 h following injection, hydrophobic compounds in plasma were collected and separated by HPLC using a Phenomenex Luna C18 5-µ column. The DNTC formulation resulted in a 10-fold increase in the level of fenbendazole metabolites found in plasma compared to the DMSO formulation (at both doses), indicating improved systemic drug absorption. We hypothesized that such increased plasma concentrations should provide increased bioavailability of these drugs to metastatic sites. To test this hypothesis, we treated AT6.1 lung metastases-bearing mice with fenbendazole solubilized either in DMSO or in DNTC. As shown in **Figures 3D, E**, we observed a greater anti-tumor activity of the fenbendazole (FBZ) drug in extending the survival of mice when solubilized in DNTC; DMSO-FBZ vs. DNTC-FBZ, mean survival days, 40.25 days (95% CI=39.5 to 41.0 days) vs. 50.75 days (95% CI=48.4 to 53.1 days), p<0.01. Additionally, no weight loss was observed in animals (n=10) treated with DNTC-FBZ compared to vehicle-DNTC (**Supplementary Figure 1A**). These data suggest that improved drug solubility improved anti-metastatic activity in these hydrophobic agents.

### Lead Drug Candidates are Pro-Apoptotic Leading to Anti-Metastatic Activity

We further compared the differential cytotoxicity observed with our promising drugs in both highly metastatic PC-3MLN4 and AT6.1 cells relative to the less aggressive PC-3M cells. We utilized the DNTC formulation for fenbendazole, fluspirilene and clofazimine, and DMSO for suloctidil and niclosamide. All drugs tested showed greater cytotoxicity (lower ED50) for highly metastatic PC-3MLN4 cells than in PC-3M cells (**Figure 4A**). Except for clofazimine and fluspirilene, a similar pattern was observed between PC-3M and AT6.1 cells. The differential cytotoxicity observed with PC-3M and PC-3MLN4 cells correlated with drug-induced apoptosis, as indicated by annexin V staining for both early and late apoptosis markers (**Figure 4B**). Greater apoptosis was observed in treated PC-3MLN4 than in PC-3M cells; these effects were significantly reduced after treatment with the caspase-3 inhibitor (z-VAD-FMK) (**Figure 4C**).

To evaluate the selective cytotoxicity effects *in vivo*, we implanted PC-3M and PC-3MLN4 subcutaneously in nude mice, waited 14 days, and then began treatment with fenbendazole (100 mg/kg, solubilized in DNTC) three times per week for five weeks. Fenbendazole treatment resulted in 44.9% tumor volume reduction in PC-3MLN4 tumors: vehicle vs. fenbendazole, 537.1% growth (95% CI=504.3 to 569.9%) vs. 296% growth (95% CI=271.21 to 320.79); compared to 16.3% reduction in PC-3M tumors: vehicle vs. fenbendazole, 515% growth (95% CI=489 to 541%) vs. 431% growth (95% CI=404 to 457) (**Figure 4D**) (p<0.005). In addition, we also treated mice bearing Dunning rat AT6.1 lung metastasis expressing secreted Gaussia luciferase with fenbendazole for two weeks (**Figure 4E**). Using measurement of plasma luciferase activity as an indicator of overall tumor burden, we observed a significant reduction in tumor burden: vehicle vs. fenbendazole, mean 165.0 RLU (95% CI=136.9 to 193.1) vs. mean 63.76 (95% CI=52.82 to 74.7), p<0.001 (**Figure 4F**). Consistently, immunohistochemical analysis of the lungs revealed reduced Ki67 staining and increased caspase-3 staining in fenbendazole versus vehicle treated mice, indicative of reduced cell proliferation and increased apoptosis in the drug-treated tumors (**Figures 4G, H**). Ki67 labeling index: vehicle vs. fenbendazole, 142.1 (95% CI=142.8 to 143.34) vs. 131.7 (95% CI=131.45 to 131.89), p<0.0001; apoptotic index: vehicle vs. fenbendazole, 1.35 (95% CI=0.94 to 1.76) vs. 18.4 (95% CI=18.19 to 18.69), p<0.0001.

### Effects of Top Agents in Paclitaxel-Resistant Prostate Cancer Cells

Paclitaxel-based chemotherapy is frequently used to treat patients with metastatic prostate cancer who develop resistance to hormone deprivation therapy; however, they often succumb within months as a result of taxane resistance (9). Paclitaxel-resistant prostate cancer cells (PC-3TxR and DU-145TxR) responded to fenbendazole in a dose-dependent manner *in vitro*, but were refractory to paclitaxel treatment (**Figure 5A**). In fact, fenbendazole, fluspirilene and suloctidil exhibited higher potency in the paclitaxel-resistant PC-3TxR cells than in the parental PC-3 cells, as evidenced by a lower ED50 (**Figure 5B**). Interestingly, only fenbendazole demonstrated higher potency in paclitaxel-resistant DU-145TxR compared to DU-145 cells (**Figure 5B**). The observed cytotoxic activity correlated with the percent apoptosis induced in these cells (**Figure 5C**). Anti-tumor activity was further confirmed *in vivo* in a subcutaneous PC-3TxR tumor model. When the tumors reached approximately 100 mm^3^ in size, the mice were treated with paclitaxel (10 mg/kg) or fenbendazole (100 mg/kg in DNTC formulation) three times per week for three weeks. Consistent with our observations *in vitro*, the growth of paclitaxel-treated PC-3TxR tumors (mean 872.5 mm^3^, 95% CI=811.7 to 933.3 mm^3^) was significantly greater when compared to the control group (mean 421.7 mm^3^, 95% CI=371.8 to 471.6 mm^3^, p=0.002) (**Figures 5D, E**). In contrast, fenbendazole-treated tumors remained small (mean 163 mm^3^, 95% CI=146.9 to 179.1 mm^3^, p=0.006) throughout the study (**Figures 5D, E**).

### Comparison of Albendazole and Paclitaxel in Experimental Bone Metastasis Model

Among the five lead drugs identified from the *in vitro* screen, fenbendazole appears to possess a distinct anti-tumor activity against various properties of metastatic prostate cancer cells. Since the aim of this study is to develop novel therapeutic agents for prostate cancer patients with metastatic disease and fenbendazole is not currently used in humans, we tested the effects of albendazole, another anti-helminth benzimidazole compound. Like fenbendazole, albendazole shares similar mechanisms of action and has been widely used to treat parasitosis in humans (32). Interestingly, albendazole also demonstrates greater cytotoxicity in highly metastatic PC-3MLN4 and AT6.1 prostate cancer cells when compared to PC-3M cells, with lower ED50 and higher induction of apoptosis (**Supplementary Figures 2A, B**). Similarly, albendazole retains cytotoxic activity against both paclitaxel-resistant PC-3TxR and DU-145TxR cells (**Supplementary Figure 2C**), suggesting potential use of this agent in men with metastatic prostate cancer. In previous human studies, albendazole has been delivered in its native insoluble form (33). In order to improve the delivery of this agent *in vivo*, we utilized the DNTC formulation and were able to enhance the bioavailability of albendazole in mice (**Supplementary Figure 2D**).

We next compared the anti-tumor activity of albendazole with paclitaxel, a standard chemotherapy regimen for men with metastatic prostate cancer. In the AT6.1 model described above, we determined that paclitaxel at 10 mg/kg (three times per week) was the optimal dose to provide extended survival with minimal toxicity (**Supplementary Figure 3**). Both paclitaxel- and albendazole-treated mice showed increased survival relative to the vehicle-treated group in the experimental lung metastasis model; mean survival days, vehicle: 26.3 days (95% CI=25.6 to 27.2 days), paclitaxel: 34.3 days (95% CI=33.2 to 35.2 days) p=0.002, albendazole 100 mg/kg: 38.2 days (95% CI=37.3 to 39.1 days), p<0.0001 (**Figures 6A, B**). There was no significant difference in survival advantage between paclitaxel and albendazole at 100 mg/kg. However, the increased survival provided by albendazole at 250 mg/kg [mean survival days, 41 days (95% CI=40.2 to 41.8 days)] was significantly greater than that seen in the paclitaxel group at its optimal dose of 10 mg/kg, p=0.008 (**Figures 6A, B**).

We further investigated the effects of albendazole (ABZ) in a model of experimental bone colonization by injecting luciferase-expressing PC-3MLN4 cells directly into mouse tibia. Mice with confirmed bone lesions (as monitored by bioluminescent imaging) were treated with 100 mg/kg albendazole for two weeks. The albendazole-treated group showed reduced intraosseous tumor growth compared to the control (**Figure 6C**), with a significant difference in average luciferase signal over the course of treatment: vehicle vs. ABZ, normalized RLU at day 15, 473.8 RLU (95% CI=390.1 to 55.8 RLU) vs. 111.5 RLU (95% CI=79.6 to 149.3 RLU), p=0.008 (**Figure 6D**). Extensive osteolysis due to the osteoclastic activity of PC-3MLN4 cells (34) was evident in the vehicle-treated mice using X-ray and micro-computed tomography (CT) imaging, whereas bone integrity was maintained in albendazole-treated mice (**Figure 6E**, **Supplementary Figures 4A, B**). Immunohistochemical analysis of these bone lesions further demonstrated a reduction of Ki-67 labeling index: vehicle vs. ABZ, mean 178.2 (95% CI=176.2 to 180.2) vs. mean 140.4 (95% CI=138.4 to 142.4), p<0.0001 and an increase in apoptotic index: vehicle vs. ABZ, mean 24.9 (95% CI=24.0 to 25.9) vs. mean 41.2 (95% CI=29.1 to 33.3), p=0.006, (**Supplementary Figure 4C**). These results indicate that albendazole has anti-tumor effects beyond the primary tumor environment and extend to disseminated tumor cells growing in intraosseous spaces.

In the therapeutic studies performed above, we were able to deliver albendazole to metastatic lesions in mice using DNTC formulation *via* intraperitoneal administration. This approach, however, may not be feasible for use in humans due to use of organic solvents. To further our attempt to develop albendazole as a potential therapeutic agent in treating men with disseminated prostate cancer, we sought a new vehicle for its systemic administration. We tested a PLGA-based method for delivering albendazole (35). Using this method, we further increased the cytotoxicity of albendazole in metastatic PC-3MLN4 as well as AT6.1 cells *in vitro* (**Supplementary Figure 5C**). When tested in the AT6.1 experimental lung metastasis model, we observed a significant survival increase in the nanoparticle albendazole (NP-ABZ)-treated group: mean survival days, 33.5 days (95% CI=30.5 to 36.5 days) relative to the vehicle-treated group (mean survival days, 23.1 days (95% CI=21.25 to 24.9 days), p<0.001 (**Figures 6F, G**). This observation is particularly intriguing as the albendazole dose used was noticeably lower than with DNTC, and there were no signs of vehicle-induced toxicity observed in treated mice (**Supplementary Figure 5**).

## Discussion

Although it is well established that tumor metastasis is the principal cause of cancer mortality, screening for cancer drugs too rarely focuses on the treatment of established metastases. Drugs that are selected for their ability to repress growth of the primary tumor or for their ability to block the dissemination of metastatic colonies are often presumed to be active against established metastases. This is infrequently found to be the case when these agents are brought to clinical trial, where they are often employed in late-stage patients. The current study was designed to find potential cytotoxic FDA-approved drugs that are not currently used for cancer but could be used to treat highly metastatic prostate cancer cells relative to less metastatic cells. Our results suggest that such a screen is feasible and show further that such drugs are effective at reducing metastatic tumor burden while prolonging the lives of these experimental animals.

This study revealed that certain drugs such as albendazole may be potent anti-cancer agents for the treatment of disseminated, advanced prostate cancers. Identification of these drugs resulted from a phenotypic (metastasis)-based *in vitro* screening of a library of marketed drugs. “Hits” from this screen exhibit greater cytotoxicity for two highly metastatic human prostate cancer cell lines relative to their less metastatic counterparts and minimal cytotoxicity on normal prostate epithelial cells. These agents were then screened in a stringent assay for a significant survival benefit in mice bearing pre-existing lung colonies of prostate cancer cells. Fenbendazole, in particular, demonstrated greater survival-promoting effects among other agents. Fenbendazole was also cytotoxic against paclitaxel-resistant prostate cancer, one of the major challenges in the clinic. Such anti-tumor effects in prostate cancer have also been observed in other members in the family of benzimidazole compounds including albendazole, oxibendazole, and mebendazole (36–38). Albendazole treatment was comparable, if not superior to, paclitaxel, a current front-line treatment for metastatic prostate cancer. We also demonstrated that albendazole was growth inhibitory to prostate cancer cells residing in lung or in bone.

The mechanism(s) by which these agents induced selective cytotoxicity on metastatic prostate cancer cells remains unclear. Niclosamide was shown to uncouple oxidative phosphorylation processes in the tapeworm and can inhibit multiple pro-survival signaling pathways in cancer cells (39, 40). Fluspirilene, a potent anti-psychotic drug, acts by blocking Ca^2+^ channels (41). While its anti-tumor activity is understudied, it was shown to promote autophagy in tumor cells without causing obvious cellular damage (42). Clofazimine, a drug for treating leprosy and tuberculosis, has also been investigated to treat colon and pancreatic cancer (43, 44). Studies showed that clofazimine inhibits cell proliferation by inducing activity of phospholipid A2 (26, 45). Suloctidil may affect cell proliferation by inhibiting cyclo-oxygenase activity, although its reported mechanism of action as a vasodilator involves inhibition of platelet aggregation (27). Finally, fenbendazole and albendazole are used commonly in veterinary and human medicine as anti-parasitic and anti-fungal agents (46), mainly by inhibiting tubulin polymerization (47, 48). Matched with our drug screen data, recent studies have also shown their anti-tumor activity in different human cancer cells (28, 37, 38, 49–51). Further studies will be required to elucidate the common mechanism(s) of action shared by these drugs (if any) in exhibiting preferential cytotoxicity towards highly metastatic prostate cancer cells when compared to the poorly metastatic cells.

Taxane-resistance has been a major clinical problem in the treatment of men with metastatic prostate cancer (10), and we are in need of alternative therapeutic options for these patients. In this study, we tested two different paclitaxel-resistant prostate cancer cell lines (PC-3TxR and DU-145TxR) in comparison to their sensitive counterparts. Despite potent cytotoxicity against metastatic prostate cancer cells, not all the lead agents identified from the screen demonstrate similar activity towards paclitaxel-resistant cells. Only fenbendazole and albendazole exhibited greater anti-tumor activity (as indicated by lower ED50) in both PC-3 and DU-145 resistant cells relative to the parental cells. Although these agents work similarly to paclitaxel by targeting microtubule polymerization, benzimidazoles may represent an alternative class of anti-cancer agents such that: 1) The tubulin-binding site of benzimidazoles is distinctly different from those targeted by paclitaxel or vinblastine; benzimidazoles bind to sites located on the outside of the microtubule while others bind near the intradimer interface on the microtubule lumen (52, 53); 2) the active metabolites of albendazole and fenbendazole are not substrates for the human breast cancer resistance protein (Bcrp1/ABCG2), multidrug resistant protein (MRP)2 or P-glycoprotein (Pgp), unlike most chemotherapeutic drugs (54).

Despite identification of fenbendazole from our screen, we determined that this veterinary compound might not be as clinically applicable when compared to albendazole, another member of the same drug family. Albendazole is mainly given orally, and it is poorly absorbed from the gastrointestinal tract due to its low aqueous solubility (34). In order to treat metastatic lesions with albendazole, efficient systemic delivery is necessary. Here, we provided the proof-of-principle evidence that improved bioavailability could lead to a greater anti-tumor activity *in vivo*. While DNTC vehicle may be applicable in experimental animals, we showed that an FDA-approved, biodegradable and biocompatible PLGA-PEG nanoparticle formulation might be more useful (and as effective) for clinical application in humans (18, 55). Our studies suggest that albendazole, when solubilized in either the improvised DNTC or nanoparticle-based formulation, exhibited profound anti-tumor activity by reducing tumor burden in the lung as well as in the bone, which consequently lead to improvement of survival of these mice. More importantly, the anti-tumor dose range for this drug was in the low micromolar range for *in vitro* experiments and 100 mg/kg for *in vivo* studies, which is within achievable pharmacological levels for humans (56). Because of its relatively benign safety profile, it should be possible to use albendazole in the long term treatment of metastatic cancers, even in patients whose tumors have become resistant to other drugs (57). A small clinical trial of albendazole in patients with advanced hepatocellular carcinoma showed promising anti-tumor activity (stabilization of disease) and well-tolerated side effects (58). A soluble derivative of the related compound, mebendazole, with potential for oral delivery was recently shown to have significant anticancer activity (59).

In summary, our phenotypic-based screening platform allows for reprioritization of drugs that are active against highly metastatic tumor cells for further clinical development. As proof of concept, we demonstrated that these drugs have profound anti-tumor activity against metastatic and taxane-resistant prostate cancer cells. Similar screens for anti-metastatic drugs should be feasible for other metastatic cancers and may reveal additional drug classes that can extend the lives of late-stage cancer patients.

## Data Availability Statement

The raw data supporting the conclusions of this article will be made available by the authors, without undue reservation.

## Ethics Statement

The animal study was reviewed and approved by Boston Children’s Hospital Institutional Animal Care and Use Committee (BCH IACUC).

## Author Contributions

All authors contributed to the article and approved the submitted version. IC and KZ contributed to drafting the work.

## Funding

The content of this article is solely the responsibility of the authors and does not necessarily represent the official views of the National Institutes of Health (NIH). Research reported in this manuscript was supported by NIH awards CA037393 (BZ), CA09381 (JB), CA155728 (DB). This study was also supported by a grant from the David Koch Foundation (BZ). The Prostate Cancer Foundation (BZ), and the Vascular Biology Program at Boston Children’s Hospital (DB).

## Conflict of Interest

A patent (application number:13/320,635, compositions for the treatment of metastatic cancer and methods of use thereof) was filed by BZ, IC, and CB, and was published on Mar 15th, 2012 (publication number: 20120064008) based on data shown in this manuscript.

The remaining authors declare that the research was conducted in the absence of any commercial or financial relationships that could be construed as a potential conflict of interest.
