# Supplementary figures and images for: Unbiased Phenotype-Based Screen Identifies Therapeutic Agents Selective for Metastatic Prostate Cancer
Source: Front Oncol. 2021 Mar 2;10:594141. doi: 10.3389/fonc.2020.594141 (PMC7962607; doi:10.3389/fonc.2020.594141)

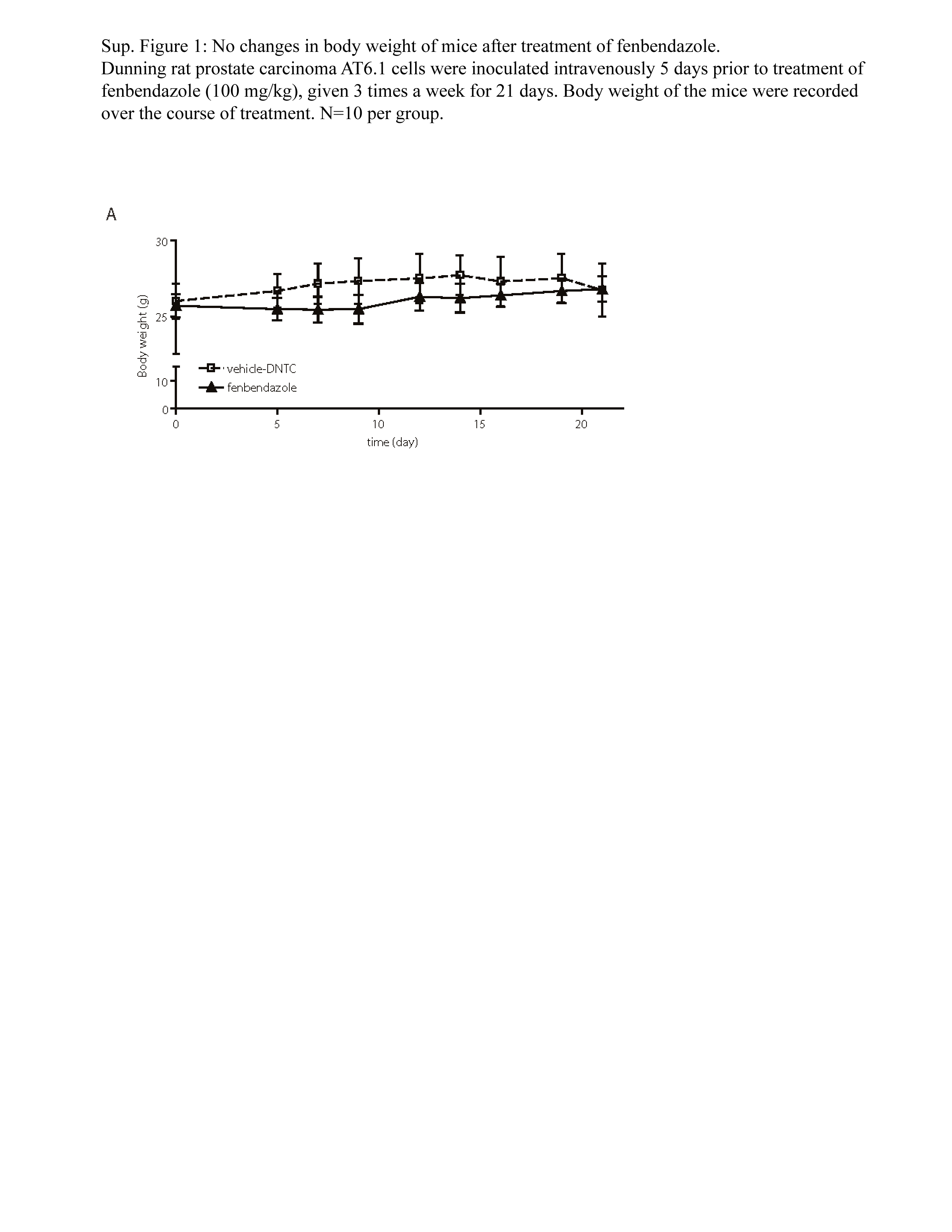

Supplement: Supplementary file 1 [file Image_1.tiff]

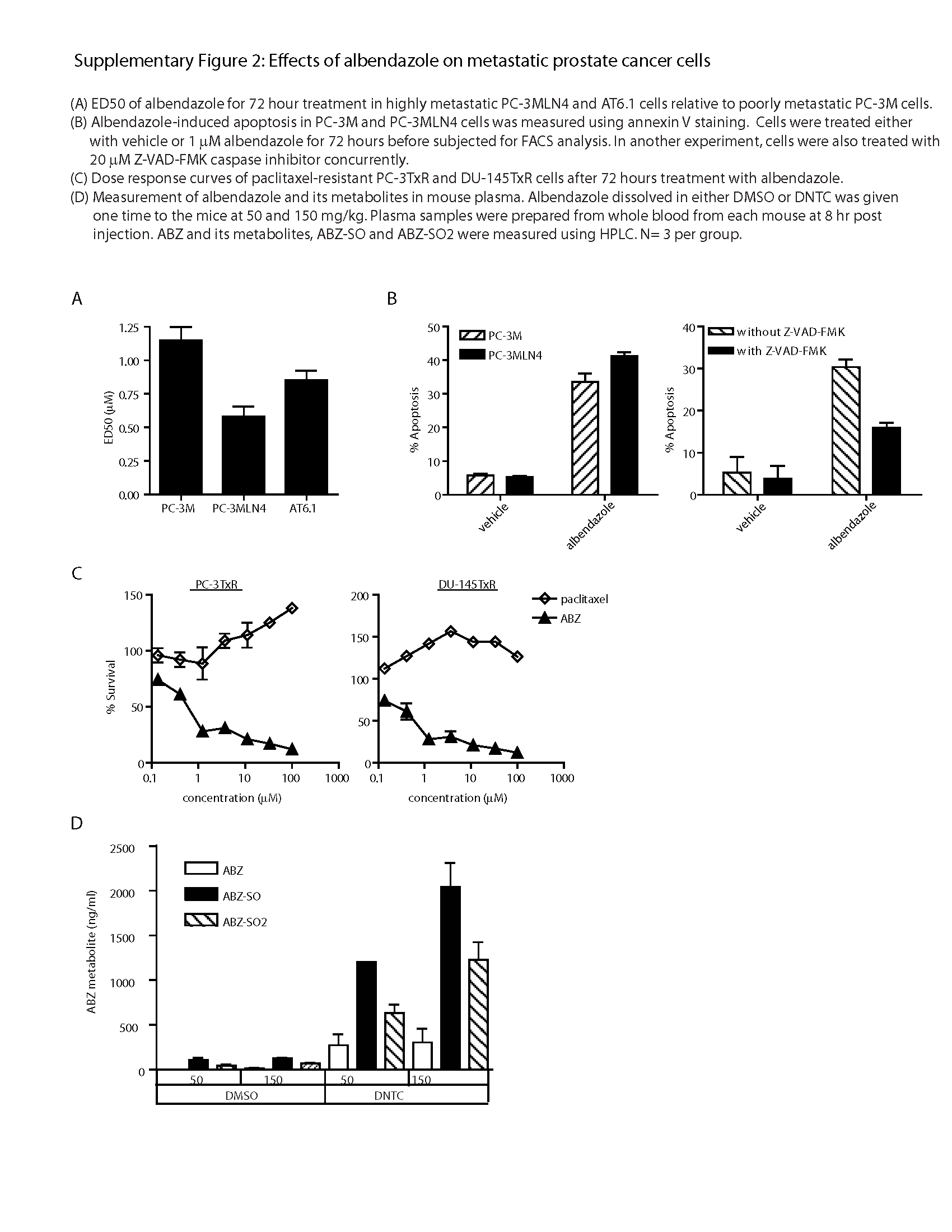

Supplement: Supplementary file 2 [file Image_2.tiff]

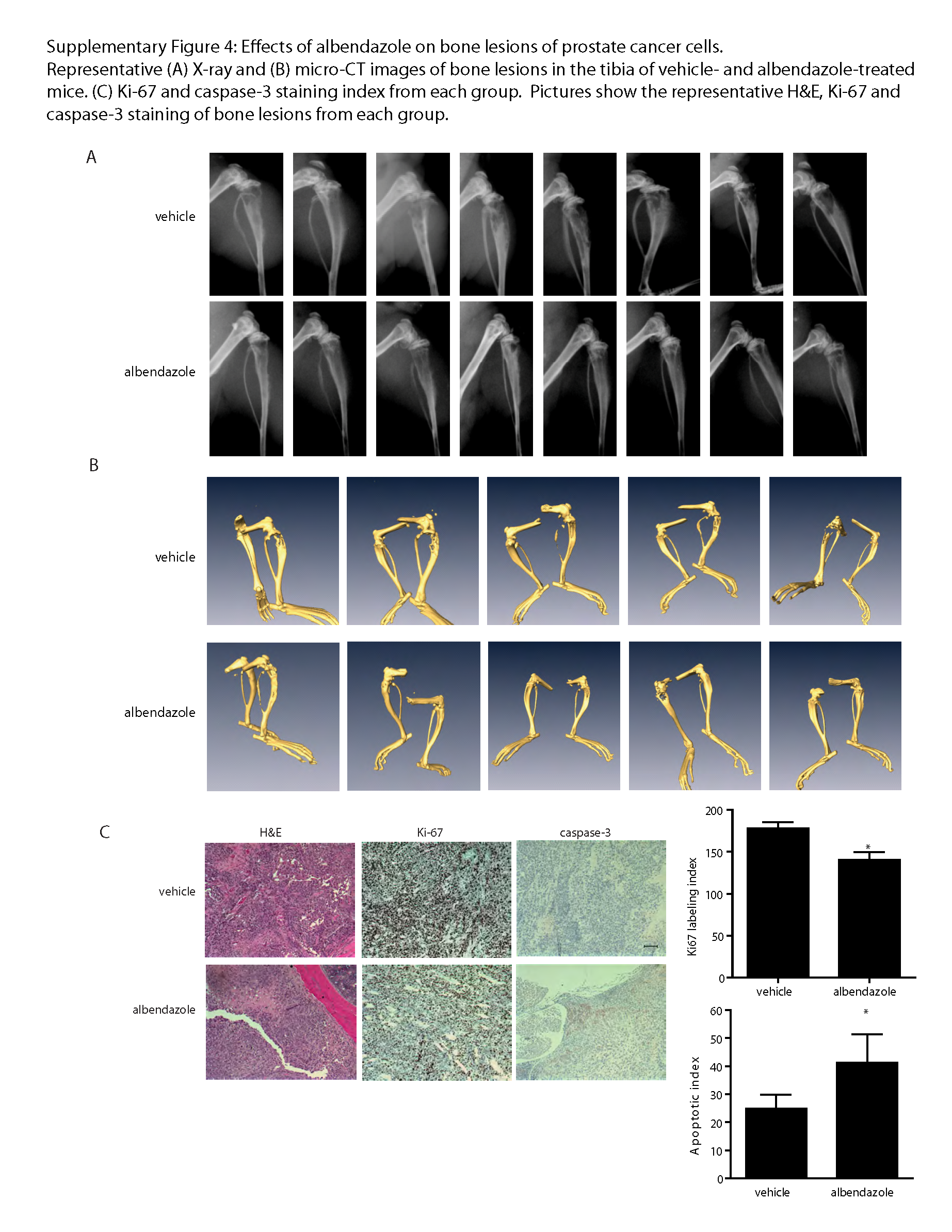

Supplement: Supplementary file 4 [file Image_4.tiff]

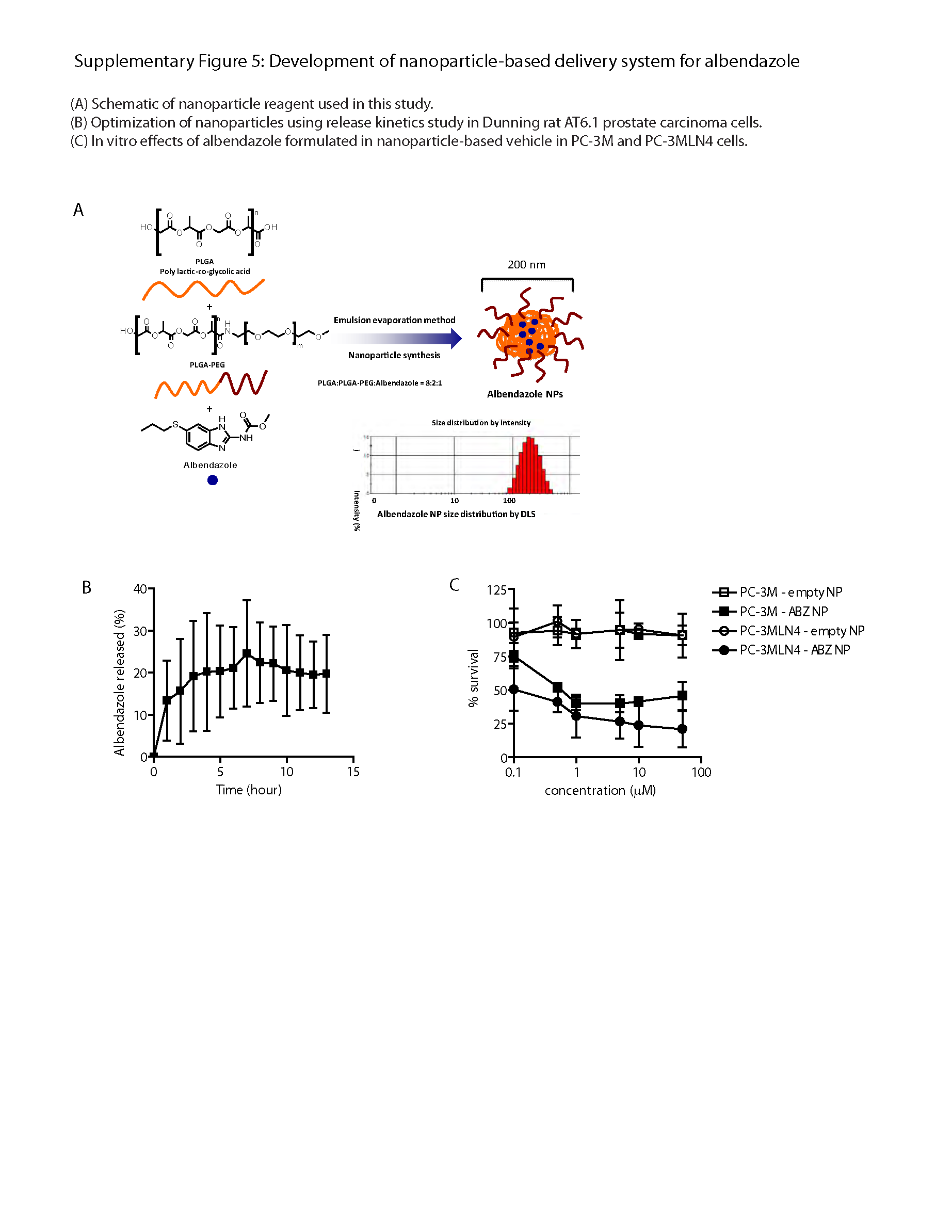

Supplement: Supplementary file 5 [file Image_5.tiff]
